# Methods of crop improvement and applications towards fortifying food security

**DOI:** 10.3389/fgeed.2023.1171969

**Published:** 2023-07-07

**Authors:** Aayushi Patel, Andrew Miles, Tara Strackhouse, Logan Cook, Sining Leng, Shrina Patel, Kelsey Klinger, Sairam Rudrabhatla, Shobha D. Potlakayala

**Affiliations:** ^1^ Penn State Harrisburg, Middletown, PA, United States; ^2^ Penn State University Park, State College, University Park, PA, United States; ^3^ Shanghai United Cell Biotechnology Co Ltd, Shanghai, China

**Keywords:** plant biotechnology, gene-editing, CRISPR/Cas9, biosecurity, sustainability, crop production, plant breeding, food applications

## Abstract

Agriculture has supported human life from the beginning of civilization, despite a plethora of biotic (pests, pathogens) and abiotic (drought, cold) stressors being exerted on the global food demand. In the past 50 years, the enhanced understanding of cellular and molecular mechanisms in plants has led to novel innovations in biotechnology, resulting in the introduction of desired genes/traits through plant genetic engineering. Targeted genome editing technologies such as Zinc-Finger Nucleases (ZFNs), Transcription Activator-Like Effector Nucleases (TALENs), and Clustered Regularly Interspaced Short Palindromic Repeats (CRISPR) have emerged as powerful tools for crop improvement. This new CRISPR technology is proving to be an efficient and straightforward process with low cost. It possesses applicability across most plant species, targets multiple genes, and is being used to engineer plant metabolic pathways to create resistance to pathogens and abiotic stressors. These novel genome editing (GE) technologies are poised to meet the UN’s sustainable development goals of “zero hunger” and “good human health and wellbeing.” These technologies could be more efficient in developing transgenic crops and aid in speeding up the regulatory approvals and risk assessments conducted by the US Departments of Agriculture (USDA), Food and Drug Administration (FDA), and Environmental Protection Agency (EPA).

## 1 Introduction

**FIGURE 1:**
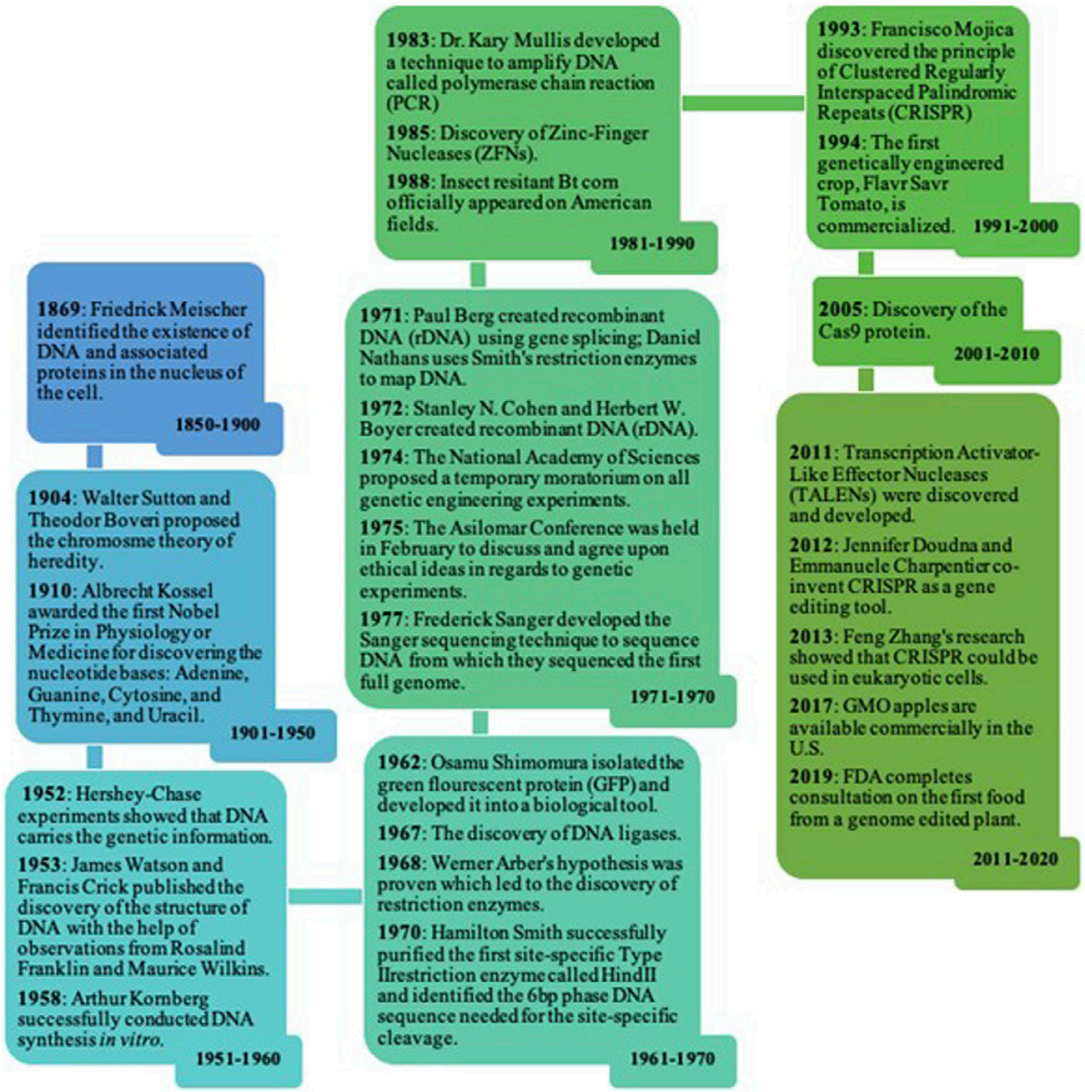
Timeline of the Advancement and Commercialization of Transgenic Crops. Many important discoveries in science led to the development of genome editing technologies that eventually lead to the advancement and commercialization of transgenic crops (U.S. Food and Drug Administration, 2022).

The year 2020 marks the 25th year of widespread cultivation of transgenic crops. In 2018 alone, 184 million hectares were dedicated to transgenic crops (Brookes and Barfoot, 2020a). Transgenic crops possess edited genomes and have been around since the late 20th century. Genome editing technologies - mechanisms by which the DNA of an organism could be edited - have advanced the field of plant biotechnology as a whole and have aided in its commercialization. However, many critical events were essential for advancing the commercialization of these transgenic crops (Figure 1). Additionally, transgenic crops have evolved and moved the ever-changing field of agriculture in a new direction. With the introduction of genome editing technologies, the field of agriculture has reached new heights through the employment of nuanced techniques in plant molecular biology and biotechnology.

Methods of crop improvement are not new, conventional breeding for a specific trait has been around since the domestication of plant species (Georges and Ray, 2017). With molecular advances, researchers began to transfer transgenes or gene elements of a known function by random integration, often achieved using *Agrobacterium*-mediated transformation (Que et al., 2010). Recombinase technology and genome engineering have both facilitated precision and targeted genomic changes for crop improvement; however, these technologies have versatile applicability in animal and plant systems (Schiml et al., 2014; Kamthan et al., 2016; Ryu et al., 2018). Due to public perception, regulation, and complexities associated with GMO animals, GE technologies have been more utilized and rapidly commercialized in plant systems (Caplan et al., 2015).

While transgenic crops work to increase the food supply’s biosecurity in the face of global change and a growing world population, there has and will be concern about the technologies use in the public forum (Husaini and Tuteja, 2013). One concern is the use of these technologies will forfeit agrobiodiversity and agronomic solutions that promote sustainability through biodiversity in the food supply. It is important to recognize that biodiversity is important; however, the use of transgenic crops and biodiversity solutions are not mutually exclusive (Jacobsen et al., 2013). In the search for novel targets for accelerated crop improvement a concerted effort can take place to query genotypes for traits, while working from a conservation genetics standpoint to preserve germplasm for research and conservation purposes.

Another concern is that these crops will hybridize with surrounding cultivars yielding economic, agronomic, or ecological consequences. Factors such as compatibility, flowering time, and spatial proximity can mitigate gene flow from pollen; however, when those factors align, gene flow is possible (Umurzokov et al., 2021). The concern for transgenes to enter native populations of phylogenetically similar plant species could lead to instances of herbicide resistance, monetarily affecting growers. Within the literature the best mode for containment remains physically blocking pollen spread in greenhouse systems, which is unviable for most growers. Biotechnology can work to find some solutions; one example in the *Brassica napus-Brassica rapa* system is placing transgenes on the C chromosome to prevent gene reoccurrence in backcrosses (Lu et al., 2002; Sohn et al., 2022). Furthermore, research on the mechanisms behind self-incompatibility in plants could provide novel solutions in the future (Zhang et al., 2023).

The delivery of improved crop cultivars presents a hurdle for stakeholders. Similar to the medical field, science generally moves more rapidly than delivery. Farmers that are encountering a changing climate and new incidences of pathogens in their fields need solutions. On a global scale, regulations on the deployment of genetically improved crops are patchy and there is a lack of consistency from country to country (Tachikawa and Matsuo, 2023). In the United States, the regulation of transgenic crops falls under the United States Department of Agriculture’s Animal and Plant Inspection Service (USDA-APHIS) (Kuzma and Grieger, 2020). Substantial policy revisions in May 2020 under the Movement of Organisms Modified or Produced Through Genetic Engineering rule led to changes where template-based editing would continue to be APHIS regulated, but point mutations and changes made that replicate features found within the plant’s natural gene pool would no longer be regulated (Department of Agriculture, 2020). For this the purposes of this review any artificial changes made to an organism’s genome is synonymous with the use of genetic engineering.

Policy and regulation require definitions and under the 2020 revision to the APHIS, a plant is considered genetically engineering by use of methods that “use recombinant, synthesized, or amplified nucleic acids to modify or create a genome” (Federal Register, 2020). While calling a plant genetically engineered is based on ‘what’ is happening to the genome, its regulation is based on the methodology behind the creation of the individual plant or cultivar.

Similarly, the EU’s recent ruling by the Court of Justice of the European Union in 2018 puts plants produce by GE methods under the regulation of GMO products. This ruling has been accessed as harming the delivery of novel cultivars and hindering the EU’s economic advantage on the world stage (Cision, 2016; Chenet et al., 2019; Wesseler and Purnhagen, 2020). The commercialization of crops produced by GE follows a stepwise processes of risk assessment followed by risk management. For the European model this is overseen by the European Commission and EU Member States. The definition of a GMO within the EU is a “an organism, with the exception of human beings, in which the genetic material has been altered in a way that does not occur naturally by mating and/or natural recombination” (Purnhagen and Wesseler, 2021).

Ethically, this begs into question stakeholder equity in the global agricultural marketplace for deployment GE products. With ununiform regulations the accessibility of improved cultivars will not be obtainable to all growers, leading some to entering a growing season with a significant financial advantage. Companies, researchers, stakeholders, and the public have an unequivocal role in advocating to lawmakers on the best practices that do not forfeit safety but fortify agricultural biosecurity.

The review article below delivers a summary of the technologies used for crop improvement followed by an outline of five unique areas researchers are working to improve in crop systems. The five areas represent individual stakeholder and grower deficits in which the discussed technologies could facilitate a higher yield and/or quality of product. Finally, the final sections expand on the challenges, advances, and industrialization within crop improvement research. This review provides an overview of transgenic crops and GE technologies while also showing how these technologies have advanced the field of plant biology. The biotechnological strides aim to tackle the problems of world hunger and sustainability.

## 2 Use of genome editing technologies in relation to transgenic crops

With respect to transgenic crops, the field of agriculture possesses a dynamic rhythm and demands constant evolution of scientific advances. Resulting from this innate characteristic of the agricultural field, GE technologies are being used and constantly improved to better society.

### 2.1 Current genome editing technologies

**FIGURE 2:**
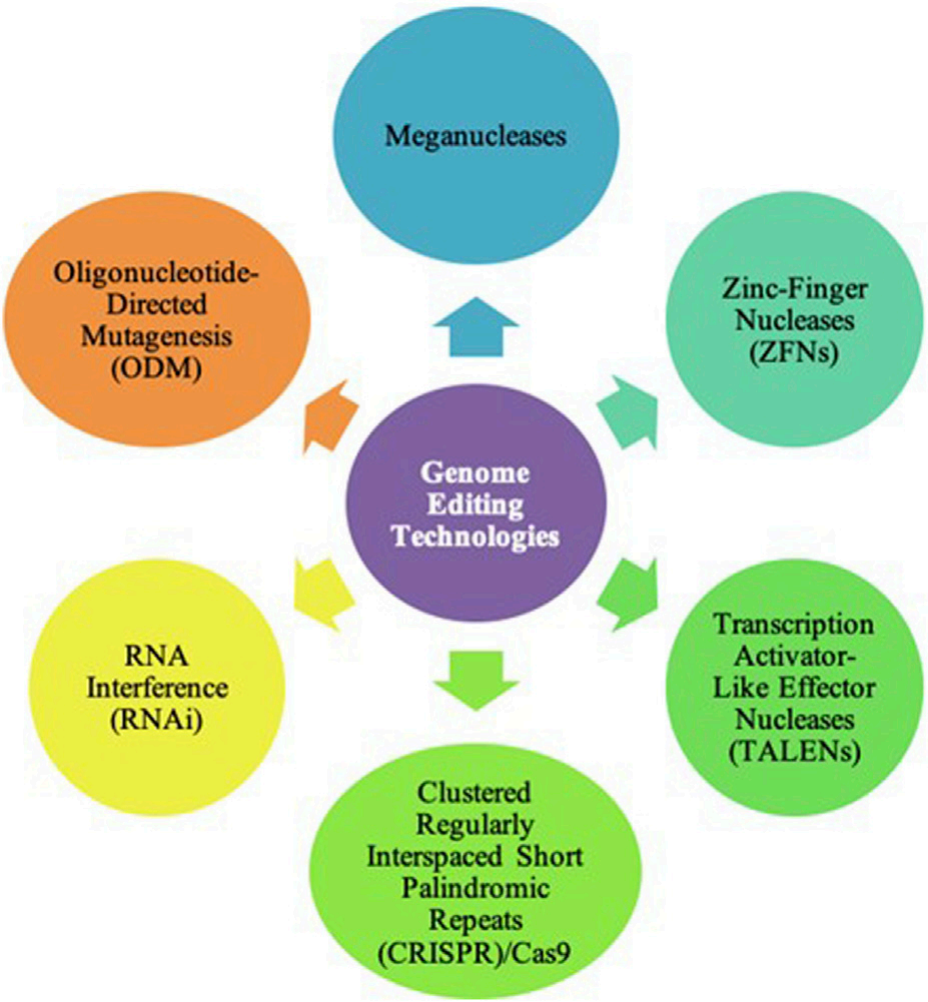
Genome Editing Technologies. To date, there are many genome editing technologies used in the field of agriculture. These include meganucleases, Zinc-Finger Nucleases (ZFNs), Transcription Activator-Like Effector Nucleases (TALENs), Clustered Regularly Interspaced Short Palindromic Repeats (CRISPR)/Cas9, and Oligonucleotide-Directed Mutagenesis (ODM). However, the most common technologies are ZFNs, TALENs, and CRISPR/Cas9.

Currently, many GE technologies are being used in the field of agriculture. The most used technologies are Zinc-Finger Nucleases (ZFNs), Transcription Activator-Like Effector Nucleases (TALENs), and Clustered Regularly Interspaced Short Palindromic Repeats (CRISPR)/Cas9 System. Other GE technologies used in agriculture include meganucleases and Oligonucleotide-Directed Mutagenesis (ODM). Additionally, RNA interference (RNAi) is not a genome editing technology but is able to improve crop characteristics without editing the genome by regulating gene expression. Figure 2 highlights the major GE technologies used in the field of agriculture.

#### 2.1.1 Meganucleases

Meganucleases, also known as homing endonucleases, were the first type of site-specific enzymes to be used in GE. Meganucleases have combined functional domains capable of binding to DNA and introducing double-stranded breaks (DSBs) in the targeted molecule. This class of restriction endonucleases are used in their natural form and is restricted to 10–40 base pair (bp) target DNA sequences (Siegl et al., 2010; Silva et al., 2011; Stoddard, 2011; Carroll, 2017).

One of the first meganucleases isolated from *Saccharomyces cerevisiae* was an intron-encoded endonuclease I (I-SceI) which could target an 18 bp sequence of DNA and is used to make DSBs (Stoddard, 2005; Takeuchi et al., 2011). Unlike I-SceI, most meganucleases are not able to function efficiently across multiple kingdoms. For this reason, meganucleases, for plant GE, are isolated from species within the plant kingdom. An example of a meganuclease isolated from a plant is intron-encoded endonuclease I from *Chlamydomonas reinhardtii* (I-CreI) has been used to make DSBs in multiple plant species (Jurica and Monnat, 1998; Gao et al., 2010; Antunes et al., 2012).

The disadvantage of this technology is that it can only target a limited number of base-pair sequences. This limitation could be overcome by using protein engineering technology. However, protein engineering technology can be time-consuming, and therefore most GE technologies like ZFNs and TALENs make use of the restriction enzyme I from *Flavobacterium okeanokoites* (FokI). Another limitation is that the combined functional domain limits the nucleases’ versatility to edit genomes across various species (Silva et al., 2011; Thyme et al., 2013).

#### 2.1.2 Zinc-finger nucleases (ZFNs)

Using zinc-finger nucleases (ZFNs) is a technique that was developed in 1990, which allowed gene editing to occur in the laboratory using a nuclease targeting a specific sequence in the DNA. The ZFNs are composed of zinc-finger-based DNA-recognition domains as well as the DNA-cleavage domain of the FokI restriction enzyme (Pabo et al., 2001; Mani et al., 2005a; Carroll, 2011; Palpant and Dudzinski, 2013). Each zinc-finger domain recognizes and binds to a nucleotide triplet, with the modules being in groups to bind to site-specific sequences. The ZFNs allow the targeting of DNA sequences that range from 6 to 18 bp (Elrod-Erickson and Pabo, 1999; Mani et al., 2005b; Gupta et al., 2012; Petolino, 2015). Unfortunately, the process requires a new nuclease each time to target a different portion of the DNA of interest. This has proven to be expensive and time-consuming, making it suitable only for one genetic modification at a time and making it challenging to study a trait associated with multiple genes (Mani et al., 2005a; Durai et al., 2005; Cathomen and Joung, 2008).

The ZFNs were effective means to develop transgenic crops such as tobacco, maize, petunia, soybean, rapeseed, rice, apple, and fig (Martínez-Fortún et al., 2017). Site-specific nucleases, like ZFNs, allow the addition of several genes into the genome of a crop in a way that allows a minimal risk of segregation. Transgene integration using ZFNs resulted in trait stacking in maize, allowing a greater potential for crop improvement (Ainley et al., 2013).

Additionally, ZFNs have been used to identify gene integration regions, gene insertion, and trait stacking in rice contributing to crop enhancement by assembling an array of valuable characteristics (Cantos et al., 2014). The technique has been employed to create double-strand DNA breaks at endogenous loci in maize while showing such breaks along with pre-integrated sequences in tobacco (Martínez-Fortún et al., 2017; Ran et al., 2017). This method has proved to be quite complicated and challenging with low efficacy, making it more efficient to use other GE tools. Furthermore, specific genes cannot be targeted using ZFNs because of the context-dependency of the ZFNs and the requirement of specific triplet DNA sequences.

#### 2.1.3 Transcription Activator-Like Effector Nucleases (TALENs)

Approximately 11 years after discovering ZFNs, the more easily accessible Transcription Activator-Like Effector Nucleases (TALENs) were developed (Romer et al., 2007; Boch et al., 2009; Joung and Sander, 2013; Jankele and Svoboda, 2014; Bucchini and Goldman, 2022). The TALENs are supposed to help target specific DNA sequences, but they are quite challenging to use in a laboratory due to their enormous size which makes it more difficult when multiple TALENs must be used simultaneously (Ellis et al., 2013; Aouida et al., 2014; Čermák et al., 2015).

The transcriptional activator-like effector (TALE) proteins were discovered in *Xanthomonas*, a plant pathogen that can target specific DNA sequences (Bonas et al., 1989; Boch and Bonas, 2010). The TALENs are fusion proteins similar to ZFNs. They are composed of TALE repeats and the FokI restriction enzyme. Each TALE repeat is bound to a target which is a single nucleotide in a DNA sequence. This allows for a more flexible target design while increasing the number of target sites possible when compared to ZFNs (Schornack et al., 2006; Boch et al., 2009; Gaj et al., 2013; Nemudryi et al., 2014).

The TALENs are a beneficial technology for crop improvement, and they have had broad applicability across several plant species, including crops such as barley, potato, tomato, sugarcane, flax, rapeseed, soybean, rice, maize, and wheat (Martínez-Fortún et al., 2017; Ran et al., 2017). The first crop that was edited using TALEN technology was rice in which the gene *Oryza sativa sucrose-efflux transporter family14* (Os*sweet14*) was disrupted, resulting in transgenic rice that was resistant to bacterial blight caused by the pathogen *Xanthomonas oryzae* (Li et al., 2012).

Additionally, TALENs have been used to modify the nutritional profiles of transgenic crops. For example, by disrupting the *fatty acid desaturase* (*fad*) genes in soybeans, transgenic soybeans containing low linoleic acid and high oleic acid were developed (Du et al., 2016). Consequently, this has helped improve the heat stability and shelf life of soybean oil (Haun et al., 2014; Demorest et al., 2016). Crop flavor has been modified by TALEN-mediated gene-editing technology, as made evident by flavor rice (Sun et al., 2013; Shan et al., 2015; Kelliher et al., 2017). Unfortunately, the development of TALE repeats is a challenge, and inconsistencies were found in gene targeting efficiency using TALENs.

#### 2.1.4 CRISPR/Cas9 system

Both ZFN and TALEN technologies displayed several issues with practical applications, but this was quickly solved with a new tool for GE. This improved technology was developed 3 years after the development of TALENs. In CRISPR, the Cas protein associates with a single guide-RNA. The complex then latches onto the complementary sequence in the genome. Finally, the Cas-RNA complex cuts the DNA at the directed site, prior to downstream modification such as insertions or deletions. The discovery of this system led to a paradigm shift in GE technologies and created a demand and a point of focus for RNA-guided nucleases. For CRISPR/Cas9 to function, RNA, specifically sgRNA, must be present, as it informs where the DNA will be edited; without this sgRNA, CRISPR/Cas9 is incapable of operating. Within the CRISPR/Cas9 system, there are different subtypes, with the most prominent and versatile genome-editing tool being the type II CRISPR/SpCas9 system which was developed from*Streptococcus pyogenes* (Jinek et al., 2012; Cong et al., 2013; Doudna and Charpentier, 2014; Hsu et al., 2014).

Unlike ZFNs and TALENs, CRISPR technology is easier to use in various models and can be used in multiple sections of the DNA. Also, CRISPR/Cas9 technology is simple, efficient, inexpensive, and can target multiple genes. The CRISPR technology made it possible to remove or silence a gene within weeks instead of decades for previous technologies. CRISPR technology can guide the insertion of DNA specifically into the cell instead of a random insertion. This technology can signal DNA repair mechanisms within the cell to arrive and assist at the location of where the DNA will be edited through the insertion of foreign DNA (Cong et al., 2013; Nekrasov et al., 2013; Shan et al., 2013; Gao, 2018). CRISPR technology has been found to have a wide array of applications including crop improvement (Pramanik et al., 2021).

The CRISPR/Cas9 technology has been utilized to create transgenic crops such as rice, maize, wheat, soybean, barley, sorghum, potato, tomato, flax, rapeseed, *Camelina*, cotton, cucumber, lettuce, grapes, grapefruit, apple, oranges, and watermelon by introducing gene knockouts (Zhang et al., 2016c; Ricroch et al., 2017). This gene knock-out method has been one of the most common methods adopted within plant biology research; it introduces small indels to cause a frameshift mutation or to introduce a premature stop codon (Liu et al., 2017).

Additionally, CRISPR/Cas9 has been used to develop herbicide resistance. By disrupting DNA ligase 4, herbicide-resistant rice was developed (Endo and Mikami, 2016). Other examples of crop-based utilization of this technology were the development of herbicide-tolerant rice using two sgRNAs targeting the repair template, the non-homologous end-joining (NHEJ)-mediated intron targeting, and the use of chimeric single guided RNA (cgRNAs) that carry both target site and repair template sequences (Li et al., 2016a; Sun et al., 2016; Butt et al., 2017). Using particle bombardment and CRISPR/Cas9 conjointly led to herbicide-resistant soybean and maize (Li et al., 2015; Svitashev et al., 2015). Herbicide-resistant potatoes were developed with the use of gemini virus replicons in combination with this system and a repair template (Butler et al., 2016). Using single-stranded oligonucleotides and the CRISPR/Cas9 system, herbicide-resistant flax was developed (Sauer et al., 2016). Glyphosate tolerance in cassava was achieved at the 5-enol pyruvyl shikimate-3-phosphate synthase (epsps) locus through a promoter swap and dual amino acid substitution (Hummel et al., 2018). All these examples represent the breadth of the applicability and the numerous advances in agronomically important crops that stem from CRISPR and the coupling of CRISPR with other technologies.

Not only is CRISPR/Cas9 effective in generating herbicide-resistant transgenic crops, but it was used to develop drought-resistant maize (Shi et al., 2017). Additionally, CRISPR/Cas9 technology has been used to develop resistance to a variety of pathogens. The technological system was used to dislocate the coding region of *Citrus sinensis lateral organ boundary 1* (Cs*lob1*), creating canker-resistant Duncan grapefruits that showed no signs of canker and Wanjincheng oranges with enhanced resistance to canker (Jia et al., 2015; Jia et al., 2017; Peng et al., 2017). Cucumber plants immune to Ipomovirus were developed by disrupting the *eukaryotic translation initiation factor 4E* (*eif4E*) (Chandrasekaran et al., 2016).

The CRISPR/Cas9 system has been used in various vegetables and fruits to knock out a gene related to the enzyme polyphenol oxidase (PPO), which causes browning, yielding economic advantages to producers (Waltz, 2016). The system was used in tomatoes to cause rapid flowering by mutating *self-pruning 5G* (*sp5G*) (Klap et al., 2017; Soyk et al., 2017). The CRISPR/Cas9 system has been influential in the development of resistance to biotic stresses in transgenic crops. The technology was used to modify three homoeologs of *enhanced disease resistance1* (*edr1*) to produce *T. aestivum edr1* (Ta*edr1*) wheat plants that were resistant to mildew (Zhang et al., 2017).

In rice, it was used to develop resistance to blast disease by targeting the *O. sativa ethylene response factor* (Os*erf922*) gene and blight resistance by targeting *O. sativa sucrose-efflux transporter family13* (Os*sweet13*) (Zhou et al., 2015; Wang et al., 2016). In tomatoes, *Solanum lycopersicum mildew resistance locus O1* (Sl*mlo1*) was targeted to develop mildew resistance, and speck resistance was developed by targeting and *disrupting S. lycopersicum jasmonate-ZIM domain2* (Sl*jaz2*) (Nekrasov et al., 2017; Ortigosa et al., 2018). The technology was also used to knockout the *thermosensitive genic male-sterile five* gene (*tms5*) in maize, resulting in thermosensitive male-sterile maize (Li et al., 2017; Li et al., 2018a). Haploid rice was also developed using CRISPR/Cas9 to knockout the *O. sativa matrilineal* (Os*matl*) gene (Yao et al., 2018).

Additionally, in rice, it was also used to knock out the *lazy1* gene–*lazy* is the representative nomenclature for the negative gravitropic response of roots resulting in the lazy phenotype–which resulted in a tiller-spreading phenotype that can increase crop yield in ideal conditions (Miao et al., 2013). The system was also used to mutate *grain number 1a (gn1a)*, *dense and erect panicle (dep1)*, and *grain size3 (gs3)* genes in rice, resulting in transgenic crops with a higher grain number, dense panicles, and larger grain size (Li et al., 2016b). The CRISPR system was used to disrupt the *Grain Weight 2* (*gw2*) gene in wheat to increase grain weight and protein content (Zhang et al., 2018). The CRISPR/Cas9 system can also be used to improve the nutritional profile of a plant. In *Camelina sativa*, the technology targeted *fatty acid desaturase gene 2* (*fad2*) to improve the oleic acid content and decrease the polyunsaturated fatty acid content (Jiang et al., 2017; Li et al., 2018b). The system targeted *starch-branching enzyme IIb (sbeIIb)* gene in rice to generate longer chains in amylopectin, which enhanced the structure and nutritional properties of starch (Sun et al., 2017). In maize, the technology was used to knockout *waxy1* (*wx1*), which encodes for granule-bound starch synthase (GBSS) responsible for amylose production (DuPont, 2022). This resulted in hybrid plants with high amylopectin content with improved digestibility while showing promise for bio-industrial applications and potential for commercialization (DuPont, 2022). The *granule-bound starch synthase* (*gbss*) gene is also being investigated using CRISPR/Cas9 technology in other crops such as potatoes, making it possible for an industrial starch market to exist (Andersson et al., 2017).

The CRISPR/Cas9 technology was also used to domesticate crops by introducing desirable traits. By targeting the coding sequences, cis-regulatory regions, and upstream open reading frames of genes, researchers domesticated wild tomatoes without selective breeding across generations. This was achieved by introducing desirable traits pertaining to tomato morphology, flower and fruit production, and ascorbic acid synthesis (Li et al., 2018c).

Most early research in CRISPR technology focused on its association with Cas9 creating the CRISPR/Cas9 system. Research showed that though the addition of this technology resulted in technological enhancement in GE technologies, the CRISPR/Cas9 system had its own share of limitations. The system showed limitations in the ability to target adenine-thymine (AT) rich regions in the DNA, in the protospacer adjacent motif (PAM), and possessing a high frequency of off-target effects (Hsu et al., 2013; Bortesi and Fischer, 2015). The CRISPR/Cpfl system is a variant created to overcome the limitations of CRISPR using a protein from *Prevotella* and *Francisella1* (Cpf1). It recognizes thymine-rich PAMs and generates sticky ends that have four or five nucleotide overhangs, unlike the blunt end breaks found in the original system. This system can also be used in conjunction with base editing or DNA-free GE technology to be completely successful (Zetsche et al., 2015).

#### 2.1.5 Oligonucleotide-Directed Mutagenesis (ODM)

Oligonucleotide-Directed Mutagenesis (ODM) is a genome-editing technique that edits the genome using synthetic oligonucleotides. ODM functions by a single-stranded sequence complementary to one strand of the DNA with minor differences, making the process non-transgenic. This process is used frequently in plant biotechnology and through this technique, a few nucleotides can be introduced to the genome using a plasmid template. The cell’s DNA repair system corrects the mismatched pairing in the synthetic oligonucleotide once it has fused with the host cell’s DNA. The process does not require expressing foreign proteins in the cell, limiting off-target effects (Papaioannou et al., 2012). This process has been used to edit the genomes of a variety of plants. The *acetolactate synthase* (*aLs*) gene in plants was first discovered through this process (Sauer et al., 2016). This ODM method is effective only if simple genomic changes are required, such as insertions, deletions, and substitutions (Sauer et al., 2016). Limitations of ODM include low correction rates and inability to control the editing process that is dependent on the oligonucleotide that is introduced.

### 2.2 Novel technical breakthroughs

Like all fields in biotechnology, GE technologies are dynamic and are evolving rapidly, especially those associated with agriculture. Recent literature has shown novel breakthroughs in biotechnology such as base editing, plastid genome and synthetic genomics, DNA-free GE systems.

#### 2.2.1 Base editing

Studies have found that single base changes are behind variations in elite traits in crops, pointing towards a need to have a GE technology that produces point mutations (Henikoff and Comai, 2003). One novel base editing technology is CRISPR/Cas9-mediated base-editing technology which accurately converts one base into another without using a DNA repair template. The CRISPR/Cas9-mediated base-editing technology requires either Cas9 nickase (nCas9) or dead Cas9 (dCas9) combined with an enzyme that can conduct base conversion activity (Komor et al., 2016). The two possible enzymes being studied are cytidine deaminase and adenine deaminase. Cytidine deaminase converts cytosine to uracil. Uracil is then treated as thymine in subsequent DNA processes. This creates cytosine-guanine to thymine-adenine substitution, giving rise to cytidine-deaminase-mediated base editing (CBE: Nishida et al., 2016). The CBE technology has been used to edit the genomes of rice, wheat, maize, and tomato. Recently, CBE technology was used to develop transgenic watermelon and wheat that are herbicide-resistant (Hess et al., 2017; Zhang and Gao, 2017; Tian et al., 2018; Zong et al., 2018). It is also known that CBE technology can generate nonsense mutations leading to gene knockouts (Billon et al., 2017).

Another class of enzymes that could be combined to form the base editing complex are adenine deaminases, which convert adenine to inosine. Inosine is then treated as guanine by polymerases. This creates an adenine-thymine to guanine-cytosine substitution which gives rise to adenine-deaminase-mediated base editing (ABE). This technique is more complicated than CBE (Nishida et al., 2016; Gaudelli et al., 2017; Lu and Zhu, 2017; Zong et al., 2017). Adenine base editors (ABEs) can convert adenine-thymine to guanine-cytosine in bacteria and humans. This process has not been applied to plants yet but could become a potential research focus in the coming years. ABEs are known to have high degrees of accuracy and purity. They are known to generate point mutations more efficiently compared to those conducted by the Cas9 nuclease. Additionally, ABEs are known to have high product purity with low indel rates and fewer off-target mutations (Gaudelli et al., 2017). This GE technique can be used to edit plant genomes to advance transgenic crops if more research focuses on expanding this technology.

#### 2.2.2 Plastid genome and synthetic genomics

Due to their smaller size, plastid genomes are the ideal platform for synthetic genomics and synthetic biology research. Their size allows the genome to be easily edited, utilizing DNA repair systems that are present within the genome. Plasmid genome and synthetic genomics have been established in multiple model organisms (Verhounig et al., 2010; Lu et al., 2013). Additionally, research shows that foreign material can be incorporated into the plastid genome, and the genome can accept and incorporate enormous quantities of genome content (ElghabiRuf and Bock, 2011; Scharff and Bock, 2014; Daniell et al., 2016). If this technique is researched adequately, it is likely to develop into an attractive GE technology.

#### 2.2.3 DNA-free genome editing systems

DNA-free GE systems are a new GE technology that produces genetically edited crops with less risk of undesirable off-target mutations in comparison to transgenic technology (Lawrenson et al., 2015; Shan et al., 2015). This technology is possible due to both protoplast-mediated transformation and particle bombardment. This method has been used in tobacco, lettuce, rice, grape, and apple (Woo et al., 2015; Malnoy et al., 2016). Particle bombardment-mediated DNA-free GE technology has been used in wheat and maize extensively (Svitashev et al., 2016). In wheat, a combination of both base editing and DNA-free GE has been used and developed (Zhang et al., 2016a; Liang et al., 2017; Zong et al., 2018). If this combination is developed and commercialized as one technique, it will work to facilitate the application of base editing to plant breeding and the commercialization of transgenic plants.

## 3 Agronomic traits and transgenic crops

The utilization of transgenic crops, especially those produced using GE technologies, has resulted in economic and environmental benefits. Benefits include increased crop yield, reduced carbon dioxide emission, increased farmer income, and improved consumer health (Klumper and Qaim, 2014; Zhang et al., 2016b; Brookes and Barfoot, 2020a; Brookes and Barfoot, 2020b). Herbicide-tolerant crops have gained many agronomic traits other than a general resistance to pests. One example is the ability to increase net yield while maintaining resistance to pests. The introduction of stacked traits, made possible using GE technologies, has advanced the agronomic characteristics of herbicide-tolerant crops and transgenic crops in general revolutionizing the market for both producers and consumers (Brookes and Barfoot, 2020a). Insect-resistant crops have introduced beneficial agronomic traits to crops by improving yield gains and providing an alternative to insecticides, which can cause unintended deleterious consequences to the environment (Brookes and Barfoot, 2020b).

## 4 Transgenic crops classifications

**FIGURE 3:**
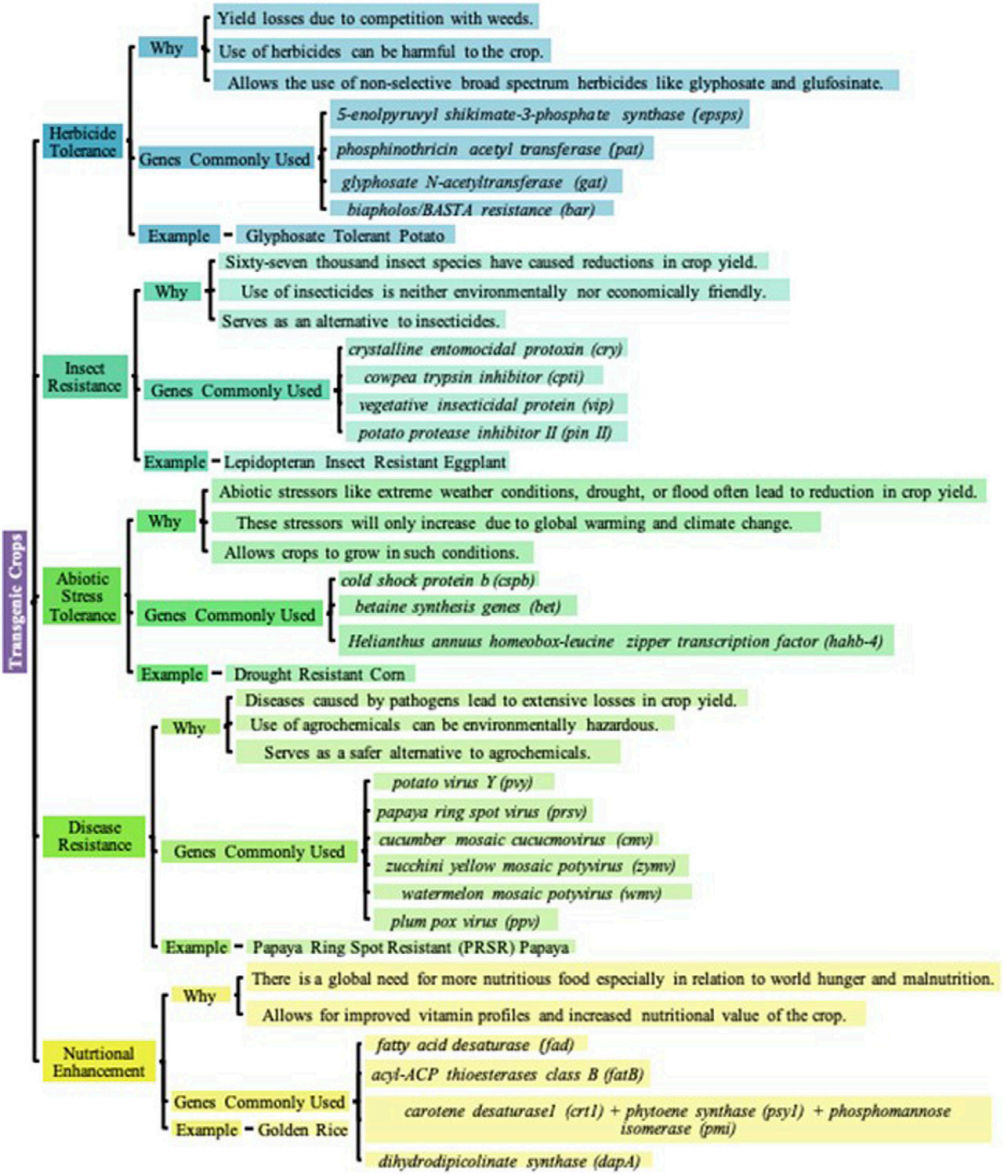
Classifications of Transgenic Crops. Transgenic crops, economically important plants that have their genomes edited often with foreign DNA, have been developed for the betterment of agriculture, increased crop yield, and crop improvement in general. Over the years, the 5 distinct categories of transgenic crops that have been commercialized are Herbicide-Tolerance, Insect-Resistance, Abiotic-Stress-Tolerance, Disease-Resistance, and Nutritional Enhancement in Crops (Yamamoto and McLaughlin, 1981; Geliebter et al., 1983; Broadway and Duffey, 1986; Hilder et al., 1987; McPherson et al., 1988; Perlak et al., 1991; Marc and Dennis, 1995; Padgette et al., 1995; Duan et al., 1996; Wehrmann et al., 1996; Takabe et al., 1998; Ye et al., 2000; Schönbrunn et al., 2001; Chen and Murata, 2002; Ferreira et al., 2002; St-Onge and Jones, 2003; Rizhsky et al., 2004; Al-Babili and Beyer, 2005; Paine et al., 2005; Ariel et al., 2007; Fang et al., 2007; Gao et al., 2007; Yan et al., 2007; Castiglioni et al., 2008; Dill et al., 2008; Kereša et al., 2008; Ufaz and Galili, 2008; Khan et al., 2009; Food and Agriculture Organization of the United Nations, 2010; Majeed et al., 2011; Pollegioni et al., 2011; Green, 2012; Rahman et al., 2012; Tuteja et al., 2012; James, 2013; Rasmussen et al., 2013; Gupta et al., 2015; Singh et al., 2015; Brookes and Barfoot, 2017; Perotti et al., 2017; Brookes and Barfoot, 2018; Raza et al., 2019; Brookes and Barfoot, 2020a; ISAAA, 2020a; Brookes and Barfoot, 2020b; ISAAA, 2020b; Kumar et al., 2020; Brookes, 2022; ISAAA, 2022; World Health Organization, 2023).

Advances in research have given way to five distinct categories of transgenic crops that have been commercialized: Herbicide-Tolerant, Insect Resistant, Abiotic-Stress-Tolerant, Disease-Resistant, and Nutritionally Enhanced Crops as shown in Figure 3.

### 4.1 Herbicide-tolerant transgenic crops

Yield losses occur globally due to the competition between weeds and crops for nutrients, water, sunlight, and space. This led to the development of herbicides which are known to have harmful side effects on the crops creating the need for herbicide-tolerant crops achievable by transgenic crops developed using GE technologies. The herbicide-tolerant transgenic crops are the most abundant commercialized transgenic crops. Herbicide-tolerant crops, in most cases, develop tolerance against non-selective broad-spectrum herbicides. Specifically, most of the herbicide-tolerant transgenic crops are tolerant to glyphosate (Brookes and Barfoot, 2020a; Kumar et al., 2020). The development of herbicide-tolerant crops allows for the use of non-selective and broad-spectrum herbicides. The two most used non-selective herbicides are glyphosate and glufosinate. Since these two are the most common, most herbicide-tolerant crops are known to be tolerant to these two herbicides (ISAAA, 2022).

Glyphosate inhibits the EPSPS enzyme. EPSPS is an essential biomolecule in the shikimate pathway of aromatic amino acid biosynthesis. This pathway is not present in the animal kingdom’s organisms, and therefore, is not harmful to animals (Schönbrunn et al., 2001). Heterologous expression of a glyphosate-insensitive form of EPSPS originating from either *Agrobacterium tumefaciens* strain CP4, a mutant maize EPSPS, or a chemically synthesize gene similar to the *epsps glyphosate resistance gene23 (grg23)* gene of *Arthobacter globiformis* plays a critical role in the development of many glyphosate-tolerant transgenic crops (Padgette et al., 1995).

The first herbicide-tolerant transgenic crop to be commercialized was the glyphosate-tolerant “Roundup Ready” soybean in 1996. This transgenic soybean had the *cp4epsps* gene, and most of the other commercialized glyphosate-tolerant transgenic crops possess this gene (Dill et al., 2008; Brookes and Barfoot, 2018). Additionally, a small number of transgenic crops can also express glyphosate oxidoreductase (GOX) or glyphosate acetyltransferase (GAT). The *GOX* gene is obtained from *Ochrobactrum etaopic*, and the *gat* gene comes from *Bacillus licheniformis*. Both genes result in the expression of glyphosate-degrading enzymes involved in glyphosate detoxification (Pollegioni et al., 2011; ISAAA, 2022).

Glufosinate, also known as phosphinothricin, works competitively to inhibit the glutamine synthetase enzyme. The glutamine synthetase enzyme is vital in the conversion of glutamate and ammonia into glutamine. If this enzyme is inhibited, then ammonia will accumulate, which will inhibit the photosystem I and photosystem II reactions. The two bacterial genes crucial in the development of glufosinate tolerant crops are *pat* genes and *biapholos/BASTA resistance (bar)* genes from *Streptomyces spp*. These genes encode phosphinothricin acetyltransferase (PAT), an enzyme that detoxifies glufosinate through acetylation (Wehrmann et al., 1996; ISAAA, 2020a).

There are also herbicide-tolerant transgenic crops that possess resistance to other herbicides such as 2,4-D, dicamba, isoxaflutole, mesotrione, oxynil, and sulfonylurea. As of 2022, there are 358 herbicide-tolerant events that have been approved for cultivation as shown in Table 1 (ISAAA, 2022).

**TABLE 1:** Herbicide Tolerant Crops. Different genetic modification events in a diverse number of crops have led to herbicide-tolerant commercial traits in different transgenic crops (ISAAA, 2022).

Crop name	Number of events leading to modification for herbicide tolerance
Maize - Zea mays L.	215
Cotton - Gossypium hirsutum L.	45
Argentine Canola - Brassica napus	37
Soybean - Glycine max L.	35
Alfalfa - Medicago sativa	4
Carnation - Dianthus caryophyllus	4
Polish canola - Brassica rapa	4
Potato - Solanum tuberosum L.	4
Chicory - Cichorium intybus	3
Sugar Beet - Beta vulgaris	3
Creeping Bentgrass - Agrostis stolonifera	1
Flax - Linum usitatissimum L.	1
Tobacco - Nicotiana tabacum L.	1
Wheat - *Triticum aestivum*	1

The benefits of commercializing and cultivating herbicide-tolerant transgenic crops include an increase in crop yield and a reduction in weed management costs. Additionally, growing herbicide-tolerant crops has reduced the environmental impact of weed management. The herbicide-tolerant crops have led to the switch to minimum or no-tillage production systems which leads to lower greenhouse gas emissions. With new aims in agriculture towards higher sustainability standards, these crops will aid in that effort by lowering machinery usage (Green, 2012; Brookes and Barfoot, 2017; Brookes and Barfoot, 2020b).

### 4.2 Insect-resistant transgenic crops

To date, 67,000 insect species have caused damage to crops that have been deemed to be economically important. Insects cause damage to a crop by draining the sap and by consuming plant tissue. Additionally, insects can be carriers of plant pathogens. To limit the loss in crop yield by insects, farmers use insecticides that are expensive and chemically synthesized. The use of insecticides is neither environmentally friendly nor economically efficient for farmers. The development of insect-resistant transgenic crops helps avoid the use of insecticides (Rahman et al., 2012; Brookes and Barfoot, 2017; Brookes and Barfoot, 2020a; Kumar et al., 2020). Table 2 shows 307 insect-resistance events that have been approved for cultivation (ISAAA, 2022).

**TABLE 2:** Insect Resistant Crops. Different genetic modification events in a diverse number of crops have led to insect-resistant commercial traits in different transgenic crops (ISAAA, 2022).

Crop name	Number of events leading to modification for insect resistance
Maize - Zea mays L.	210
Cotton - Gossypium hirsutum L.	50
Potato - Solanum tuberosum L.	30
Soybean - Glycine max L.	6
Rice - Oryza sativa L.	3
Sugarcane - Saccharum sp	3
Poplar - Populus sp.	2
Cowpea - Vigna unguiculata	1
Eggplant - Solanum melongena	1
Tomato - Lycopersicon esculentum	1

There are ten commercialized insect-resistant transgenic crops. For genetic transformation to develop insect-resistant transgenic crops, insecticidal genes, generally variants of the *crystalline entomocidal protoxin (cry)* genes and *vegetative insecticidal protein (vip)* genes, are used. Insect-resistant transgenic crops are the second-largest transgenic crop category. Insect resistance has been developed in maize, cotton, potato, soybean, rice, sugarcane, poplar, cowpea, eggplant, and tomato (Keeše, 2008; ISAAA, 2022).

The *cry* genes from *Bacillus thuringiensis* are often used to develop insect resistance and are non-toxic to mammals. This gene produces the CRY protein, which helps form crystalline inclusions in bacterial spores resulting in insecticidal activities. The CRY toxin fragment has three domains. The first domain results in pore formation. The second domain helps in receptor binding, and the third domain is involved with protecting the CRY toxin from proteases. The toxin fragment will bind to specific receptors, courtesy of the second domain. The first domain will then insert itself leading to pore formation in the cell membrane of the insect’s epithelial cells which leads to insect paralysis and death (Yamamoto and Mclaughlin, 1981; McPherson et al., 1988).

The *cry* genes have been known to provide resistance to multiple orders of insect pests such as lepidopterans, coleopterans, and dipterans. The *cry* genes are involved with gene stacking resulting in a more stable insect resistance capability by the transgenic crop. The first commercially successful insect-resistant transgenic crop that had *cry* genes was cotton, and it was resistant to insects in the taxonomic order of lepidopteron (Perlak et al., 1991). The *cry* genes have been added to the genomes of potato, rice, canola, soybean, maize, chickpea, alfalfa, and tomato (Kumar et al., 2020).

The *vip* class of genes isolated from the *Bacillus* species, which express vegetative insecticidal proteins, has also been used. Heterogeneous expression of *vip3A(a)* is possible in cotton, while heterogeneous expression of *vip3Aa20* is found in maize (Fang et al., 2007; ISAAA, 2022).

Another source to develop insect resistance in transgenic crops is through *protease inhibitor* (*PI*) encoding genes derived from various sources. The PI proteins are defense-related proteins in plants that are employed in response to physical injury such as an insect attack. The PIs inhibit insects’ proteolytic enzymes, making it difficult for insects to acquire the amino acids required for their growth and development (Broadway and Duffey, 1986). Two specific PIs, potato protease inhibitor II and trypsin inhibitor, are known to inhibit insect digestive enzymes. These two PI genes have been incorporated into transgenic rice, cotton, and tobacco to enhance their insect resistance. Specifically, the *potato protease inhibitor II (pinII)* gene was incorporated into rice and cotton, while the *trypsin inhibitor (ti)* was incorporated into tobacco (Hilder et al., 1987; Duan et al., 1996; Majeed et al., 2011). Currently, three events involving PIs have been approved: in cotton, the *cowpea trypsin inhibitor (cptI)* gene from *Vigna unguiculata*, was introduced, the *arrowhead protease inhibitor (api)* gene in poplar was introduced from *Sagittaria sagittifolia*, and the *pinII* gene from *Solanum tuberosum* was introduced into maize (ISAAA, 2022).

### 4.3 Abiotic-stress-tolerant transgenic crops

Environmental factors, known as abiotic stressors, are not ideal for crops and can negatively impact the crop’s growth and development, which eventually leads to a loss in crop yield. Abiotic stressors include but are not limited to drought, extreme temperatures, heat, cold, flooding, and salinity. In the current state of global warming and climate change, the impact of abiotic stressors on crops has increased significantly and will only continue to rise (Tuteja et al., 2012). High throughput phenotyping and advances in bioinformatics allows researchers to screen for key genomic features that contribute to abiotic stress tolerance. The combined approach of using single nucleotide polymorphisms (SNPs) in tandem with genome-wide association studies (GWAS) can provide a set of features to modify for crop improvement (Thoen et al., 2017; Oladzad et al., 2019). The importance of these technologies within the context of crop improvement and transgenics is a result of the direct application of the knowledge to GE. Precision in choosing quality gene targets is a deliverable of the advances made in bioinformatics. Furthermore, the integration of machine learning and deep learning predictive models in trait improvement can assist in preventing off-target modifications when deploying gene-editing technologies (Charlier et al., 2021).

Traits conferring tolerance to abiotic stressors can be found in landrace cultivars of crops; these plants are a bountiful resource for genetic diversity. Landrace cultivars have adapted to unique abiotic conditions in regions all over the world. These cultivars are molded by generations of natural and artificial selection for enhanced growth and yield in niche environments (Dwivedi et al., 2016; Casañas et al., 2017; Yolcu et al., 2020). The bioinformatic tools can act to screen the genomes of landrace cultivars to sequester a subset of candidate targets for gene editing. The methodology of using genomic and bioinformatic resources for stress tolerance is a common practice for plant breeders; however, to facilitate faster commercialization for end-users the methodology can be applied in a gene-editing framework (Sharma et al., 2022).

One example, a landrace rice cultivar from Bangladesh was found to have traits for salinity tolerance with a specific QTL related to the sodium-potassium ratio (Rahman et al., 2019). Furthermore, in the face of global change, brought and salinity are two stressors requiring advances in cultivar performance for growers. Wheat landraces from Mexico were characterized for drought and salinity tolerance where accessions were flagged as performing positively under stress. The study highlights the ability to utilize cultivars of a staple food growing in niche environments for downstream GMO applications (Suhalia et al., 2022).

Along with most GMO technologies, the use of landraces for commercialization of new cultivars poses ethical concerns. Many of the landrace farmers are indigenous people and their ownership of the cultivars is generally not patented. This begs the question as if and how much these groups should be compensated. Furthermore, the larger question of ‘who owns biodiversity’, becomes an ethical dilemma (Gepts, 2004). Additionally, this type of research highlights the necessity for landrace conservation as a long-term investment in the genomic resource for abiotic stress tolerance (Poudel and Johnsen, 2009; Mathew and Mathew, 2023). The use of genetic editing technologies discussed above allows for researchers and companies 1.) to bring new cultivars with abiotic tolerance to market quicker and 2.) to prevent a reshuffling and reorganization of alleles during outcrossing to cultivars with novel phenotypes (Fasoulas, 1980).

To survive under conditions that consist of growing levels of abiotic stress, plants alter their metabolism. This is usually done by activating signal cascades and regulatory proteins such as transcription factors and heat shock factors which activate and modify the antioxidant defense system (Rizhsky et al., 2004). This response acts to help maintain homeostasis and synthesize and accumulate compatible solutes such as polyamines, sugars, betaines, and prolines that help maintain osmotic homeostasis in plants. These adaptations to abiotic stressors allow the crops to experience minimal adverse effects, which is achieved by maintaining plant growth and development conditions close to optimal (Gao et al., 2007). Abiotic stressors can also result in alterations in gene expression, which then calls for the interplay of multiple gene networks to maintain near-optimal conditions for growth, development, and survival (Rasmussen et al., 2013; Gupta et al., 2015; Raza et al., 2019; Kumar et al., 2020).

Compared to herbicide tolerance and insect resistance, not as many abiotic stress tolerance events have been approved and commercialized due to the process’s innate complexity. Currently, thirteen events have been approved and commercialized globally as shown in Table 3 (ISAAA, 2022).

**TABLE 3:** Crops Resistant to Abiotic Stress. Different genetic modification events in a diverse number of crops have led to commercial traits for resistance to abiotic stressors in different transgenic crops (ISAAA, 2022).

Crop name	Number of events leading to modification for resistance to abiotic stress
Maize - Zea mays L.	7
Sugarcane - Saccharum sp	3
Soybean - Glycine max L.	2
Wheat - *Triticum aestivum*	1

Extreme temperatures can have an impact on plants and yield and can impact plant physiology such as photosynthesis, growth, and nutrient content. One KO gene target of the CRISPR/Cas system, *OsPRP1*, improves the sensitivity to extreme cold temperatures in rice (Li et al., 2022). The gene codes for a proline-rich protein (PRP) that is most likely a cell wall protein; PRPs are associated with conferring abiotic and biotic stress tolerance. The likely mode of function of the gene is that it plays a role in the modulation of antioxidants and expression of genes for other pathways (Nawaz et al., 2019). Plants have a complex network of signaling pathways, with interplay and crosstalk between multiple pathways for a myriad of stressors under the control of a single gene (Ben Rejeb et al., 2014). Due to the confounded complexity of plant signaling networks, the effects genome edits make must be thoroughly validated for unintended effects. Hence, validation through a multi-omics approach can be a powerful method to confirm only intended changes are made to the system (Qian and Huang, 2020).

Often, bacterial cold shock proteins (CSPs) are used to develop abiotic stress tolerance in transgenic crops. Cold shock proteins are a group of bacterial RNA chaperones known to resolve misfolded RNA structures into stable forms, which contributes to maintaining cellular functions in dehydration conditions. Specifically, *cold shock protein (csp)* genes, *cold shock protein a (cspA)* from *Escherichia coli* and *cold shock protein b (cspB)* from *Bacillus subtilis*, have been introduced to crops to develop cold, heat, and water-deficit rice or maize. A study also showed that using *csp* for the development of transgenic crops did not have pleiotropic effects. The water-deficit maize was better adapted to water-deficient conditions, but it was still able to grow under optimal watering conditions (Castiglioni et al., 2008). In 2013, Monsanto’s drought-tolerant transgenic maize hybrids were launched in the United States for commercialization as Genuity Drought Guard. This drought-tolerant maize required the introduction of *cspB* and had a reduced water requirement. A drought-tolerant and insect-resistant maize was developed to tackle two problems that were clouding and hindering the agricultural market in the Sub-Saharan region of Africa (James, 2013; ISAAA, 2022).

Transcription factors (TFs) have also been used to develop transgenic crops tolerant to various abiotic stressors successfully. One specific class of TFs used is the homeodomain-leucine zipper (HD-Zip) class, which is explicitly found in plants. This class of TFs has highly conserved homeodomain (HD) and leucine zipper (Zip) motifs. The HD-Zip class of TFs interact with abscisic acid-regulated developmental networks (Ariel et al., 2007; Perotti et al., 2017). Another gene used to develop abiotic stress-tolerant crops is the *betaine synthesis (betA)* gene, derived from either *E. coli* or *Rhizobium meliloti*, which encodes choline dehydrogenase. Choline dehydrogenase catalyzes the formation of glycinebetaine, an osmoprotectant compound that aids in adapting to water-related stressors. Osmoprotectants protect the cell membrane and maintain the osmotic potential. Studies have shown that accumulating osmoprotectants or compatible solutes like non-reducing sugars, proline, and glycinebetaine in a plant helps a crop survive under osmotic stress (Takabe et al., 1998; Chen and Murata, 2002; Khan et al., 2009; Singh et al., 2015).

### 4.4 Disease-resistant transgenic crops

Pathogen-caused diseases are heavily prevalent and result in extensive crop loss. Plant diseases, historically, have been taken care of by agrochemicals. Due to concerns about agrochemicals being environmentally and economically hazardous and risk of developing pests resistant to agrochemicals, other means of resisting the effects of diseases had to be explored. In addition, overcoming the challenges associated with plant pathogens continues to be a significant hurdle for producers. This led to the consensus that it is vital to develop disease resistance in crop plants (Kumer et al., 2020).

As of 2022, 29 events resulting in disease-resistant transgenic crops have been approved and commercialized globally as shown in Table 4 (ISAAA, 2022).

**TABLE 4:** Disease-Resistant Crops. Different genetic modification events in a diverse number of crops have led to disease-resistant commercial traits in different transgenic crops (ISAAA, 2022).

Crop name	Number of events leading to modification for disease resistance
Potato - Solanum tuberosum L.	19
Papaya - Carica papaya	4
Squash - Cucurbita pepo	2
Bean - Phaseolus vulgaris	1
Plum - Prunus domestica	1
Sweet pepper - Capsicum annuum	1
Tomato - Lycopersicon esculentum	1

Among these crops, many are resistant to viral pathogens. Virus resistance in crops is developed through mechanisms of gene silencing. Four different approaches have been successfully used to develop viral resistance. The first approach is to express the viral *coat protein* (*cp*) gene, which results in resistance using the pathogen-derived resistance mechanism (Kumar et al., 2020). An example of this approach is clearly seen in an event in squash which has now been commercialized. In this event, the squash gained resistance against the cucumber mosaic virus, the zucchini yellow mosaic virus, and watermelon mosaic virus II. Additionally, this approach saved the papaya crop from global extinction due to the papaya ring spot virus (PRSV; ISAAA, 2022). The *cp*of PRSV was expressed in the papaya crop resulting in pathogen-derived resistance. This event was commercialized as Rainbow or SunUp Papaya (ISAAA, 2022).

The second approach is expressing a defective viral replicase or a helicase domain, resulting in resistance by gene silencing. Resistance to PRSV in papaya was developed using this approach by using the*rep* gene of PRSV to develop viral resistance and commercialized as Huanong no. One papaya. The third approach is to express sense and antisense RNA strands of the viral replication protein (REP). The fourth and final approach is to use the antisense RNA to degrade the mRNA that codes for a critical often essential viral protein (Marc and Dennis, 1995; Ferreira et al., 2002; Yan et al., 2007; Kumar et al., 2020).

### 4.5 Nutritionally improved transgenic crops

With the growing world population, there is a new and increasing need to produce enough food to sustain the human population on Earth. This can be made possible with the use of transgenic crops. Transgenic crops can not only increase crop yield but by nutritionally improving crops, farmers will be able to feed more individuals with the same amount of crop. Table 5 summarizes approved events that result in modified product quality that often results in a nutritionally improved profile. Additionally, Table 5 also shows other plants that also have modified product quality such as carnation which is not related to nutritionally improved profiles but does aid in the commercialization of the plant (ISAAA, 2022).

**TABLE 5:** Crops With Modified Product Quality. Different genetic modification events in a diverse number of crops have led to commercial traits for modifying product quality in different transgenic crops (ISAAA, 2022).

Crop name	Number of events leading to modification for modified product quality
Carnation - Dianthus caryophyllus	19
Potato - Solanum tuberosum L.	18
Maize - Zea mays L.	14
Soybean - Glycine max L.	12
Argentine Canola - Brassica napus	10
Tomato - Lycopersicon esculentum	9
Apple - Malus x Domestica	3
Alfalfa - Medicago sativa	2
Melon - Cucumis melo	2
Petunia - Petunia hybrida	2
Rice - Oryza sativa L.	2
Rose - Rosa hybrida	2
Safflower - Carthamus tinctorius L.	2
Cotton - Gossypium hirsutum L.	1
Pineapple - Ananas comosus	1
Tobacco - Nicotiana tabacum L.	1

One prominent example of a nutritionally improved transgenic crop is the provitamin A biofortified rice, known as Golden Rice. In recent times, Vitamin A deficiency (VAD) has become a public concern, mainly due to the rise in the global population and therefore increasing world hunger and malnutrition. According to the World Health Organization, an estimated 250 million preschool children experience VAD (World Health Organization, 2020). Additionally. It is estimated that 250,000 to 500,000 VAD children become blind every year, with half of them dying within the first 12 months of losing sight (World Health Organization, 2020). The VADs are heavily prevalent in developing countries.

The required precursor for vitamin A biosynthesis is beta-carotene which is not present in conventional rice. To help combat VAD, transgenic rice with provitamin A was developed. For Golden Rice to develop, two genes were used. The first gene was the*phytoene synthase (psy)* gene from daffodil, which encodes for phytoene synthase, and the second gene was the *carotene desaturase1 (crtI)* gene from *Erwinia uredovora*, which encodes for carotene/phytoene desaturase; however, this study did not produce enough carotenoid accumulation to solve the VAD problem in the world (Ye et al., 2000). It was later recognized that the *psy* transgene was the limiting factor in having a higher carotenoid content. In 2005, the *psy* gene from maize, which has higher activity and the *crtI* gene, were used to develop Golden Rice II (GR2) (Al-Babili and Beyer, 2005; Paine et al., 2005). Carotenoid accumulation was marked and noted in this rice and was deemed to be appropriate to tackle a challenge like VAD.

Oils provide many essential fatty acids. Another class of nutritionally improved transgenic crops is those that have modified oil or fatty acid content. This has been mainly accomplished to improve the nutritional qualities of seed oil because oils with a low saturated fatty acid content and a higher proportion of polyunsaturated fatty acids (PUFAs) are better for human consumption (Food and Agriculture Organization of the United Nations, 2010). This is known to particularly benefit the heart as it reduces the levels of low-density lipoproteins (LDLs) and triglycerides. Additionally, long-chain triglycerides (LCTs) are substituted with medium-chain triglycerides (MCTs), which increases the basal metabolic rate and simultaneous storage of less adipose tissue. Furthermore, higher content of monounsaturated fatty acids (MUFAs) improved both stability and flavor (Geliebter et al., 1983; St-Onge and Jones, 2003). There have been 18 events approved and commercialized that improved oil and fatty acid content (ISAAA, 2022).

The third prominent group of nutritionally improved transgenic crops alters the essential amino acid content. Certain amino acids can only be obtained through diet, and among these are lysine, tryptophan, and methionine. These amino acids are essential for biofortification. Additionally, lysine and tryptophan are not abundantly found in cereals, and methionine is not abundant in legumes. In recent years, approaches have been utilized to alter plant proteins’ amino acid composition to engineer essential amino acid metabolic pathways and increase the content of specific amino acids, leading to nutritional enhancement. An example of improving amino acid content is in transgenic maize, in which lysine and tryptophan content was improved (Ufaz and Galili, 2008). As of 2022, two events of improving amino acid content, both in maize, have been approved and commercialized (ISAAA, 2022). Additionally, there have also been two events in modifying starch and carbohydrate content as well (ISAAA, 2022).

## 5 Technological advancement of transgenic crops

Though transgenic crops have existed longer than GE technologies, GE technologies have played an instrumental role in advancing transgenic crops. GE technologies have allowed for efficient manipulation of crop genomes, leading to the advancement of transgenic crops through researchers’ ability to introduce gene modifications such as insertions, deletions, and substitutions in specific locations in the genome. This has led to crop improvement by introducing advantageous agronomic traits into the genome of crops and improving the functionality of existing genes ultimately lowering the cost for farmers while simultaneously increasing crop yield. For heightened sustainability, the crops have reduced negative environmental impacts such as greenhouse gas emissions and pesticide usage (Brookes, 2022). The change in USDA policy on biotechnological regulation has increased the objectivity in assessment and the feasibility of bringing a product to market. The revisions foster science- and risk-based policy while streamlining the process, increasing sustainability and competition by lowering the burden to commercialize a crop improved through GE (Hoffman, 2022).

### 5.1 Biological challenges

Even though GE technologies have advanced transgenic crops, there are still biological challenges exhibited by transgenic crops. One biological challenge is exhibited in herbicide-tolerant crops, especially those tolerant to glyphosate. The concern about occurrences of glyphosate-resistant weeds is a major topic for those in agriculture. This challenge exists mainly due to the use of glyphosate as the sole means of weed control, resulting from its broad-spectrum post-emergence activity putting selection pressure on weeds. The selection pressure results in weeds adapting “genes of resistance” in populations exposed to non-transient interactions (Brookes and Barfoot, 2020a).

Additionally, weeds are developing resistance against other herbicides. According to the Current Status of the International Herbicide-Resistant Weed Database, there are 514 unique cases of herbicide-resistant weeds globally, encompassing 262 weed species (Norsworthy et al., 2012). Weeds have developed resistance to 23 out of 26 known herbicide sites of action and have developed resistance against 167 different herbicides. These weeds have also been reported in 93 crops in 70 other countries (International Herbicide-Resistant Weed Database, 2022).

Alternatively, weed resistance is promoted by the adoption of no or reduced tillage production techniques. This is mainly occurring in North and South America, where such techniques are more common than in other regions of the world. Reduced or no-tillage techniques may result in a population shift towards weed species that are not controlled by glyphosate. To combat this biological challenge, cultivators of herbicide-tolerant crops are advised to rely on alternative herbicides with a different or complementary mode of action in combination with glyphosate to control weeds. This technique would reduce the pressure on the weeds to evolve resistance to glyphosate, which minimizes and slows down the process of weed resistance.

Other recommendations to reduce the risks associated with herbicide-tolerant crops have been identified (Norsworthy et al., 2012; Brookes and Barfoot, 2020b). Another solution to this biological challenge is the adaptation of stacked herbicide-tolerant transgenic crops, which are developed using GE technologies. These stacked crops offer a combination of tolerance to active ingredients and glyphosate (Brookes and Barfoot, 2020a).

Another biological challenge is resistance breakdown which occurs when there is extensive cultivation of transgenic insect-resistant or herbicide-tolerant crops. The cultivation of these transgenic crops puts selection pressure on insects and weeds. This pressure could then result in the evolution of new insect biotypes and the emergence of resistant weeds; however, genes can help delay resistance breakdown (Norsworthy et al., 2012; Bawa and Anilakumar, 2013; Gilbert, 2013).

Unfortunately, studies have reported adverse effects on non-target organisms, with monarch butterflies being at the forefront. With the adaptation of glyphosate resistance crops, the population of monarch butterflies in Mexico and the United States has decreased (Brower et al., 2012). Additionally, a shift in weed population has been reported to kill significant pests, causing secondary pests to take over (Mertens, 2008). The decrease in population is mainly due to the decline in the population of milkweed plants as a result of transgenic crops and the use of herbicides (Losey et al., 1999). However, studies show that *B. thuringiensis* (Bt) maize has little to no adverse effects on monarch butterflies and their larvae (Sears et al., 2001; Dively et al., 2004).

## 6 Commercialization of genome editing technologies

The use of GE technologies to produce transgenic crops has aided in the commercialization of these crops. The use of GE technologies has allowed for the regulatory process to speed up, making it easier for transgenic crops to be commercialized. Additionally, the United States Department of Agriculture (USDA) has judged that crops produced by technologies such as CRISPR are not to be considered Genetically Modified Organisms (GMOs; U. S. Environmental Protection Agency, 2020). Transgenic crops produced using GE technologies are often treated in the same manner as those developed through conventional breeding methods, lowering development and approval costs. The positive impacts of these crops, such as higher yield in less time and inputs, have also motivated farmers to cultivate these crops for commercialization. Overall, development of transgenic crops through GE technologies in relation to have boosted individual income and the agricultural sector, making way for a stronger economy (Brookes and Barfoot, 2020b).

### 6.1 Crop regulation

The use of GE technologies has made the process for crop approval and commercialization easier with crops developed using GE technology not considered GMOs making its acceptance and approval process much more efficient than traditional transgenic products (USDA, 2018). Specifically, USDA has decided not to regulate certain products developed from CRISPR. Also, USDA has announced that certain products developed using ZFNs and TALENs will not be under their regulatory jurisdiction (Wolt et al., 2016; USDA, 2018; USDA APHIS, 2020). This eases the ability of agribusinesses and farmers to develop, use, and commercialize transgenic products developed from GE technologies. Furthermore, crops developed using GE technologies are treated equivalent to those produced using conventional breeding methods. This classification brings about a lot of advantages for companies, farmers, and consumers while lowering development and approval costs associated with this type of regulation and allowing for the decentralization of the market.

### 6.2 Biosafety issues

There have been concerns raised that transgenic crops can be harmful to human health due to their potential toxicity, allergenicity, and their possible risk to the environment. Specifically, critics point to the possibility of transgene flow into the environment harming biodiversity (Mertens, 2008; Suzie et al., 2008). The critics of these crops have argued against the use of transgenic crops since the onset. In 1998, ‘Starlink’ maize, which expresses *crystalline entomocidal protoxin9c (cry9c)*, was not approved for human consumption in the United States because it had a substantial risk of being allergenic to humans. This risk was due to the protein’s high stability and its potential to interact with the immune system (Suzie et al., 2008). In 2000, this same transgenic maize experienced a global recall because residues of the CRY protein were detected in food products and had caused allergic reactions; however, most studies that focused on health hazards associated with the consumption and use of transgenic crops have not reported adverse effects on animal health (Domingo, 2016; Tsatsakis et al., 2017; De Vos and Swanenburg, 2018; U. S. Environmental Protection Agency, 2020). Many are also concerned that horizontal gene transfer of antibiotic-resistant genes from transgenic food to gut microflora could cause antibiotic resistance. The possibility of this occurring is extremely low, and the development of marker-free transgenic crops is rising (Heritage, 2004; Netherwood et al., 2004; Keese, 2008; Tuteja et al., 2012).

An environmental issue relating to transgenic crops is pollen-mediated transgene flow (Watrud et al., 2004; Ford et al., 2006; Han et al., 2015; Yan et al., 2015). This could result in a loss of biodiversity. There could be detrimental effects if the transgene flows to weeds and relatives of weeds, which has been documented with herbicide-resistant weeds. Herbicide-resistant weeds would not only damage the environment, but they could also damage the agricultural economy at all levels (Heap, 2014; Heap and Duke, 2018; International Herbicide-Resistant Weed Database, 2022). Pollen-mediated transgene flow poses a potential risk to organic growers as well. Those who use their crop’s seed for the subsequent growing season could be at risk of planting crops fertilized by transgenic pollen.

### 6.3 Cost of commercialization

Transgenic crops developed using GE technologies affect farm income and production positively. Each type of transgenic crop has a particular effect on the cost and profits of commercialization. Overall, the cost to farmers to use this technology for all four main crops was 27% of the total value of the gains of using technology. The total cost was 23% of the total technology gain in developing countries, while it was 31% in developed countries (Brookes and barfoot, 2020). There are two major reasons for the discrepancy in the cost between developed and developing countries. Developing countries will have weaker intellectual property rights and have a higher average farm income gain per hectare. These two reasons are the main factors playing into the discrepancy noticed between developing and developed countries (Brookes and Barfoot, 2020b).

Herbicide-tolerant crops were among the first and most prominent types of transgenic crops cultivated and commercialized on a large scale. Herbicide-tolerant crops have been proven to be a more cost-effective and easier means of weed control than other methods from conventional technology (Brookes and Barfoot, 2020a; Brookes and Barfoot, 2020b). The magnitude of these crops’ impacts, especially commercially, has been known to vary by country and by year. Several factors are known to be behind this variation as well. One such factor is the costs of different herbicides used in herbicide-tolerant crop systems compared to other weed control practices. There are specific factors that have a larger effect on the level of cost-saving compared to the cost of commercialization that could occur when using herbicide-tolerant crops for cultivation and commercialization (Brookes and Barfoot, 2020a; Brookes and Barfoot, 2020b).

The first factor is that the mix and the amounts of herbicides used are dependent upon the price and availability of herbicides. These prices can also vary year by year and country by country, based on factors including but not limited to exchange rates, costs to manufacture, and distribution (Brookes and Barfoot, 2020a; Brookes and Barfoot, 2020b). Secondly, farmers must pay to use herbicide-tolerant transgenic crops developed using GE technologies. Pricing of such technology and other forms of seed and crop protection technology is known to vary based on the level of benefits farmers could expect to receive from its use. This factor is also dependent upon intellectual property rights. Specifically, in countries with weaker intellectual rights, the cost of these technologies will be lower in comparison to countries with stronger intellectual rights (Brookes and Barfoot, 2020a). Additionally, the landscape of the types of herbicide-tolerant transgenic crops available to farmers for cultivation is changing. In the first 15–20 years of herbicide-tolerant crop cultivation, crops were dominantly glyphosate-tolerant (Brookes and Barfoot, 2020a; Brookes and Barfoot, 2020b). In 2018, farmers, especially those in North America, had the option of using seeds with stacked tolerance to glyphosate along with other active herbicide ingredients such as glufosinate, 2,4-D, and dicamba (Brookes and Barfoot, 2020a; Brookes and Barfoot, 2020b).

The third factor is the biological challenge faced by glyphosate-resistant crops. Responses to this biological challenge have affected mainly the mix, the total amount, the cost, and the overall profile of herbicides applied alongside herbicide-tolerant crops (Brookes and Barfoot, 2020a; Brookes and Barfoot, 2020b). In general, when comparing 2018 to the early 2000s, this factor has resulted in higher weed control costs associated with herbicide-tolerant crops (Brookes and Barfoot, 2020a; Brookes and Barfoot, 2020b; International Herbicide-Resistant Weed Database, 2022). Compared to conventional practices, the use of herbicide-tolerant transgenic crops still provides more economic advantages for many of its users, generally in the form of lower production costs or higher yields. This mainly occurs because herbicides used in accordance with conventional methods can still produce significant weed resistance.

Additionally, since herbicide-tolerant global crop adoption levels have not fallen in recent years, it is believed that farmers are most likely deriving important economic benefits from using this technology. From this, one can see that even though there is a cost of commercialization and cultivation associated with herbicide-tolerant crops, farmers are still receiving critical economic benefits from using herbicide-tolerant transgenic crops, leading to the technology not being reduced in use (Brookes and Barfoot, 2020a). Over the years, the benefits of commercializing and cultivating transgenic crops have outweighed the minimal costs associated with commercialization.

### 6.4 Economic benefits of commercialization

There have been many economic benefits of commercializing transgenic crops that outweigh commercialization costs by a significant amount. Overall, US farmers are the largest beneficiaries of higher incomes due to this technology’s adaptation as they witnessed 96 billion USD in extra revenue from 1996 to 2018 which is mainly due to the widespread adoption of this technology in the United States (Brookes and Barfoot, 2020a). In South America, 58.7 billion USD of farm income benefits was realized mainly from transgenic soybeans and maize (Brookes and Barfoot, 2020b).

Additionally, insect-resistant cotton has resulted in an additional 47.5 billion USD of additional income in China and India. Developing countries earned 53.7% of the global farm income benefits in 2018, and most of these benefits were from insect-resistant cotton and herbicide-tolerant soybean. From 1996 to 2018, the cumulative farm income gain was 117.1 billion USD for farmers in developing countries, equal to 52% of the total farm income in this period (Brookes and Barfoot, 2020a).

For each extra dollar invested in GM technology, farmers gained about 3.75 USD in additional income. In developing countries, the gain is about 4.41 USD, and in developed countries, it is approximately 3.24 USD (Brookes and Barfoot, 2020b). From 1996 to 2018, 72% of the total income gain was from higher yields and second-crop soybean gains, while 28% was from lower costs. Insect resistance has resulted in 56.9% of the total income gain. In comparison, herbicide tolerance has resulted in 42.9% of total income gains, and other traits such as abiotic stress resistance account for 0.2% of the total income gain. In 2018, 88% of the total income gain was due to gains in yield and production, and the remaining 12% was from cost savings. This is mainly due to the adaptation of the second generation of transgenic crops (Brookes and Barfoot, 2020a).

### 6.5 Genome editing patents and multinational companies

Genes used by GE technologies to develop transgenic crops can be patented which limits the access and research expansion for these crops. Most commercialized events have been patented, and the majority are being patented by the top five multinational companies as shown in Table 6 (Bonny, 2017; Business Wire, 2020). With the addition of joint ventures between the companies, it has become increasingly difficult for smaller firms to compete in this market. Therefore, it is difficult for farmers as well since they have fewer choices in the seed market, which raises the price of the product globally. Many ethical issues stem from this type of oligopoly of information, technology, and patents. The lobbying power of these major multinational companies creates a lot of tension with small-scale farmers and researchers.

**TABLE 6:** Top 5 Multinational Biotechnology Companies. The top 5 multination biotechnology companies in the transgenic crops market are Monsanto, Dupont, Syngenta, Groupe Limagrain, and Bayer CropScience (Raman, 2017; Bayer, 2022; Dupont, 2022; Forbes, 2022; Limagrain, 2022; Syngenta, 2022).

Company	Company net income	Global seed industry market share (%)
Monsanto	2.26 billion USD	26
Dupont	204 million USD	18.2
Syngenta	4 million USD	9.2
Groupe Limagrain	98.8 million USD	4.8
Bayer CropScience	85 million USD	3.3

### 6.6 Genome editing sector

GE technologies have boosted the agricultural sector of the economy and the gene-editing sector. In 2015, biotechnology companies received $1.2 billion in venture capital funds, about 16.3% of the corporate venture investment capital. This makes it the second-highest funded sector in the United States, with the market projected to grow with a Compound Annual Growth Rate (CAGR) of 13.75% (Market Reseach, 2020; Venture Capital, 2020). Since 2013, leading companies using CRISPR technology received over $600 million in venture capital and public market investments (Van Erp et al., 2015). In addition, it has been predicted that the market share of genome-edited seed is expected to increase by 14.63 billion USD from 2021 to 2025 (Technavio, 2022).

Experimentation has been limited to swine in recent years, but an expansion to cattle and sheep could revolutionize animal science. The recent advances in animal reproductive biology, not limited to *in vitro* fertilization, microinjection of embryos, and cloning, form the foundation for future advances in animal gene-editing technologies (Eriksson et al., 2018). Much like plant production, animal production faces challenges in a changing climate that is coupled with increasing demand, yet these technologies allow for the induction of advantageous genes into livestock genomes. The major barriers are public opinion on the topic and the complex regulatory framework (Van Eenennaam, 2019). Additionally, ethical issues surrounding the ability of small-scale farmers to keep up with large industrial farms is a contentious issue, but the technology has the potential to reshape animal-based agriculture.

## 7 Conclusion

One of the most revolutionary advances in plant biotechnology has been the ability to transform plants through *Agrobacterium*-mediated transformation. This single technique changed plant breeding and agriculture. Currently, the new gene-editing technologies within plant biology research are revolutionizing crop improvement, at a time when humanity faces many major crises. DNA-free editing shows much promise in becoming a new method of genetic engineering. This technology uses premade ribonucleoprotein complexes and nanoparticles for targeted genome modification. Furthermore, the employment of viral vectors could become a major player in pathogen resistance and genetic modification (Nouri et al., 2018; Tsanova et al., 2021). Generally, plant biotechnology utilizes plant tissue culture as a central component of the quintessential “triad of plant biotechnology,” tissue culture, transformation, and molecular biology; however, if technologies could arise to negate tissue culture, that could be a major advantage that would streamline the process (Atkins and Voytas, 2020).

Regulatory hurdles can hinder and damper the advances in plant biotechnology by delaying a product’s commercialization. Complex bureaucratic agencies and regulations for genetically modified crops stand to protect consumers from harm, but the risks of delaying a beneficial product by layers of regulatory “red tape” could have negative consequences for consumers and producers alike. Currently, plants modified using plant-derived sequences are not within the regulatory purview of the vast majority of federal agencies (Francis et al., 2017). As plant biotechnology becomes relied on more heavily in the future, in conjunction with the pressures within agriculture, the regulatory framework could see minor or major changes.

The applications of gene-editing technology have yet to be fully realized by researchers. What seemingly was viewed as science fiction less than three generations ago, humans can now fundamentally change through recent biotechnological advances. Using CRISPR/Cas9 in forestry to tackle the increasing problem of tree pathology is a major opportunity for exploration. Invasive species and diseases have fundamentally changed the forests of the Eastern United States and gene-editing technologies can offer possible solutions (Dort et al., 2020). RNA editing is another research area that could be further explored in the future. Applications of this technology could offer advances in functional gene analysis (Mishra et al., 2020). Utilizing recoded viruses to control diseases in plants has much potential. Due to the technology being transgene-free, regulatory hurdles are not encountered, and this technology has the ability to revolutionize approaches to plant pathology (Kumar and Singh, 2021). Researchers stand on the Frontier of what is considered possible, as novel discoveries and innovations unfold, achievement of sustainability, biosecurity, and the ability to feed all people become less distant realities.
